# P-1202. Human Bocavirus detection among children presenting to tertiary care with acute respiratory infections in Oman: clinical characteristics, seasonality and comparison with common viruses

**DOI:** 10.1093/ofid/ofae631.1384

**Published:** 2025-01-29

**Authors:** Ahmed S Al Fahdi, Abdulrahman S Al Qanobi, Mohammed Al Maani, Bushra Al Maani, Mariya Al Khatri, Hanan Al Khatri, Abdulaziz Al Hinai, Zaid Alhinai

**Affiliations:** College of Medicine and Health Sciences, Sultan Qaboos University, Muscat, Ad Dakhiliyah, Oman; College of Medicine and Health Sciences, Sultan Qaboos University, Muscat, Ad Dakhiliyah, Oman; College of Medicine and Health Sciences, Sultan Qaboos University, Muscat, Ad Dakhiliyah, Oman; College of Medicine and Health Sciences, Sultan Qaboos University, Muscat, Ad Dakhiliyah, Oman; College of Medicine and Health Sciences, Sultan Qaboos University, Muscat, Ad Dakhiliyah, Oman; Oman Medical Specialty Board, Ibri, Az Zahirah, Oman; College of Medicine and Health Sciences, Sultan Qaboos University, Muscat, Ad Dakhiliyah, Oman; College of Medicine & Health Sciences, Sultan Qaboos University, Muscat, Masqat, Oman

## Abstract

**Background:**

Human Bocavirus (HBoV) was first identified in 2005 among children suffering from acute respiratory symptoms. It is frequently detected as a co-infecting virus, and the degree to which it causes to symptoms is unclear. Little is known about its epidemiology in the Middle East.

**Figure:**
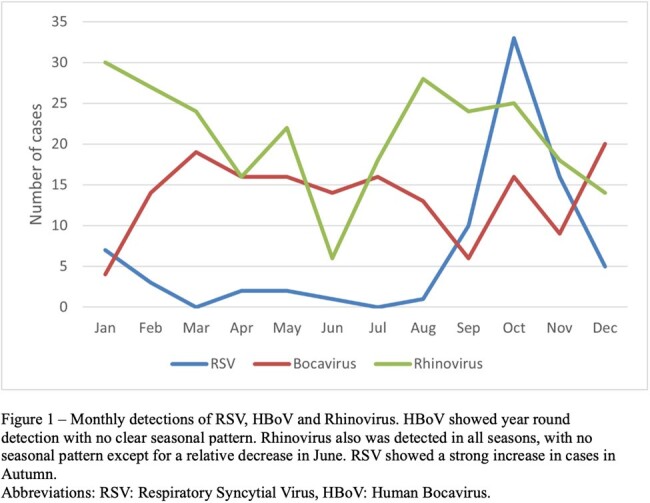

**Methods:**

Children who presented to Sultan Qaboos University Hospital, Muscat, Oman between January, 2018 and December, 2019, with acute respiratory symptoms and were tested for respiratory viruses were reviewed. Respiratory specimens were tested using the FTD Respiratory Pathogens 21 Assay (Siemens Healthineers). Patients with HBoV detection were included. A subset of patients with sole detection of RSV or Rhinovirus was included for comparison. Clinical presentation, laboratory results, and outcomes were analyzed and compared.

**Figure:**
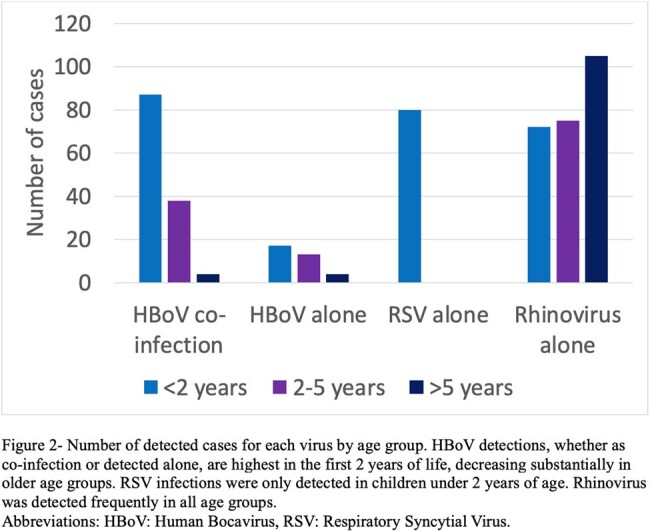

**Results:**

A total of 3789 patients were tested for respiratory viruses, of whom 2714 (72%) had positive results, and 163 (5%) were positive for HBoV. HBoV was detected as the only pathogen in 34 cases (21%), while 129 (79%) represented co-infection. For comparison, 252 cases of Rhinovirus and 80 cases of RSV were included. HBoV detection occurred year-round with no clear seasonal variation (Figure 1). HBoV detection was most common in children under 2 years of age, and uncommon after 5. RSV was only detected under 2 years of age, and Rhinovirus was commonly detected in all age groups (Figure 2). Patients with HBoV without co-infection were more likely to be immunocompromised (29% vs 8%, P 0.002) or suffer from other chronic disease (68% vs 35%, P 0.001) compared to those with co-infection (Table 1). They were also more likely to suffer from diarrhea (35% vs 14%, P 0.009) and tachycardia (77% vs 54%, P 0.032). Patients with HBoV without co-infection did not significantly differ otherwise from those with co-infection. Compared with RSV, patients with HBoV had a higher median age (1.95 vs 0.2 years, P < 0.001) and were more likely to be immunocompromised (29% vs 1%, P < 0.001) (Table 2). Patients with RSV were more likely to require respiratory support (67% vs 29%, P < 0.001).

**Figure:**
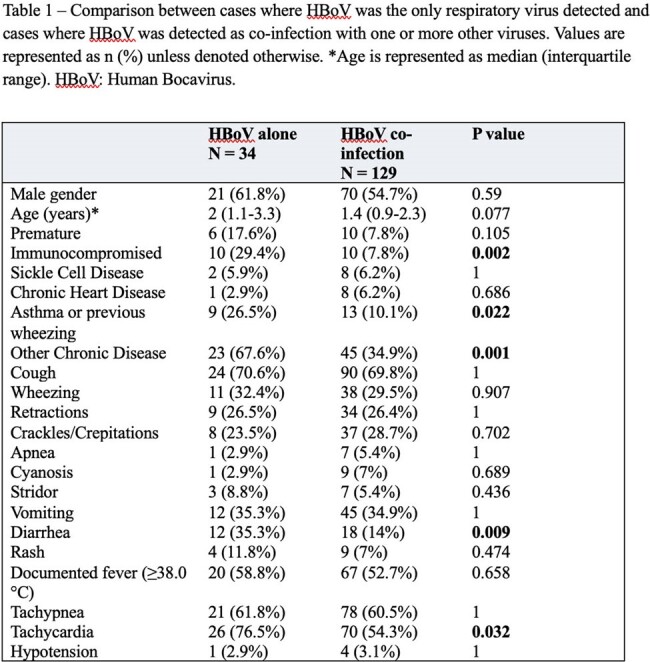

**Conclusion:**

HBoV had year-round circulation with no seasonal pattern. HBoV likely contributed significantly to acute respiratory disease in children under 5 years of age, especially those with chronic underlying conditions.

**Figure:**
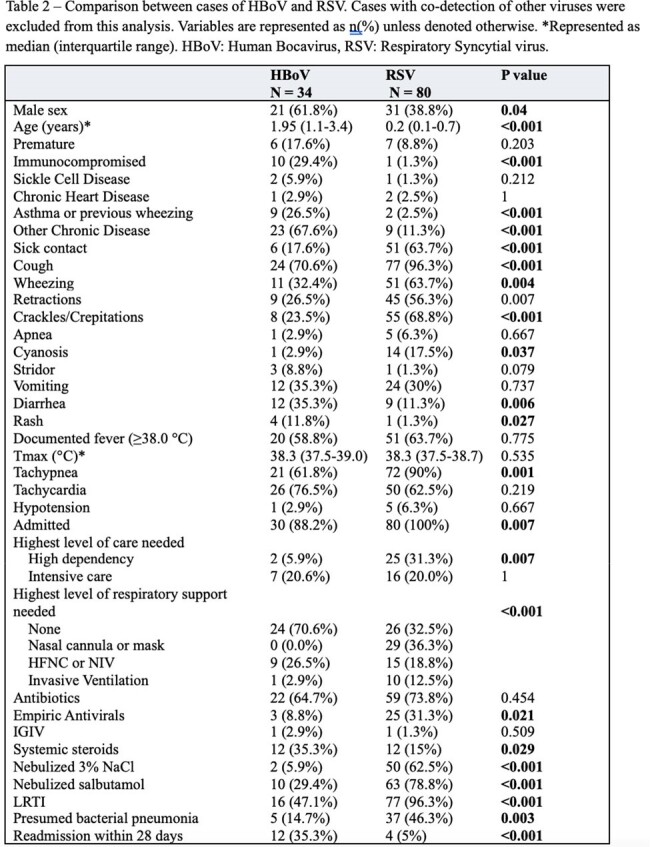

**Disclosures:**

**All Authors**: No reported disclosures

